# Correction: An open-source closed-loop Virtual Reality system to investigate social interactions and collective behavior in fish

**DOI:** 10.1371/journal.pone.0343509

**Published:** 2026-02-20

**Authors:** Stéphane Sanchez, Ramón Escobedo, Renaud Bastien, Boris Lenseigne, Audrey Denis, Mathieu Moreau, Maud Combe, Andrew D. Straw, Clément Sire, Guy Theraulaz

The affiliation for the ninth author is incorrect. Clément Sire is not affiliated with #4 but with #3: Laboratoire de Physique Théorique, CNRS, Université de Toulouse III – Paul Sabatier Toulouse, France.

In the legend of Figure S1 Video, the unit of speed is incorrectly indicated as m/s. The correct unit should be cm/s. Please view the correct S1 Video below.

## Supporting information

S1 Video Comparison of a fish’s behavior in the presence of a virtual fish and then a virtual ball under control conditions. Video excerpts of an experiment with a real fish interacting with the anamorphic projection of a virtual conspecific during 30 minutes and then with a non-social control stimulus consisting of a black sphere during the next 30 minutes in the experimental bowl of radius 250 mm. Both the virtual fish and virtual sphere move along a uniform circular trajectory with a constant speed of 10 m/s, at a constant depth of 5 cm below the water surface, and at a constant distance of 10.4 cm to the edge of the tank. The video shows, after one minute, the replacement of the three-dimensional fish model with the spherical model. Top-Left: user interface allowing the real-time visualization of the trajectories of the real and virtual fish (in the xy- and xz-planes) and the instantaneous modification of the parameters of the model driving the virtual fish. Bottom-Left: real-time 3D tracking of the real fish. Right panel: anamorphic rendering of the virtual fish projected onto the acrylic bowl by the rendering application according to the 3D position of the real fish.
(MP4)
