# Supplementary material for: An open-source closed-loop Virtual Reality system to investigate social interactions and collective behavior in fish
Source: PLoS One. 2026 Jan 21;21(1):e0339909. doi: 10.1371/journal.pone.0339909 (PMC12823003; doi:10.1371/journal.pone.0339909)
Supplement: S1 Text — (PDF) [file pone.0339909.s001.pdf]

S1 Text. Experimental setup, software architecture, and trajectory design for the closed-loop virtual reality system

1 Experimental setup diagram and parts

The experimental setup frame is made of modular aluminum strut profiles (S1 Fig). The frame dimensions (width $\times$ depth $\times$ height) are $1\,\mathrm{m} \times 1\,\mathrm{m} \times 2\,\mathrm{m}$. The fish tank top is fixed with an aluminum plate at mid-height of the frame. The depth camera is fixed (at about 50 cm above perpendicularly to the middle of the tank) with an articulated camera arm. IR filters are applied to the depth camera lenses. Eight IR lamps illuminate the tank from underneath. Each lamp is made from an IR LED with a 100 W LED heat sink and mounted on an articulated camera arm. A high-resolution, low-latency LED video projector is mounted on the frame, pointed at a mirror under the tank that reflects the projected virtual 3D scene on the tank. To prevent perturbations from external sources during experiments, three of the upper sides and the top of the frame are covered with black acrylic plates. The last side is closed with a blackout curtain that allows easy access to the fish tank and depth camera. S1 Table lists all materials and equipment used in the experimental setup.

2 Software

Closed-loop interactions between real fish and virtual ones require three software:

- Acquisition and 3D tracking software that gets frames from the Intel RealSense D435 camera and tracks fish positions from them:
  https://doi.org/10.6084/m9.figshare.30188836.v1
- Trajectory simulator that simulates virtual fish behaviors, with or without interactions with real fish:
  https://doi.org/10.6084/m9.figshare.30188842.v1
- Rendering software that displays virtual fish according to the simulated positions and real fish position (to perform anamorphosis rendering):
  https://doi.org/10.6084/m9.figshare.30188845.v1

The rendering software needs calibration routines and application:
https://doi.org/10.6084/m9.figshare.30188839.v1

3 Perimeter of a rhodonea.

In polar coordinates, the equation of a rhodonea can be written as $r(t) = R\cos(mt)$, where $m = n/d$. The arc length ds is given by

$$\begin{aligned} ds &= \sqrt{r^2 + \left(\frac{dr}{dt}\right)^2}\, dt = \sqrt{R^2\cos^2(mt) + R^2m^2\sin^2(mt)}, \\ &= R\sqrt{1 + (m^2-1)\sin^2(mt)}. \end{aligned}$$

The perimeter is thus

$$P = \int_0^{d\pi} \sqrt{r^2 + \left(\frac{dr}{dt}\right)^2}\, dt = R\int_0^{d\pi} \sqrt{1 + (m^2-1)\sin^2(mt)}\, dt.$$

Changing variables $k = mt$, we have $dt = \frac{1}{m} dk$, and when t goes from $0 \to d\pi$, then k goes from $0 \to md\pi = n\pi$. Thus,

$$P = R\frac{d}{n} \int_0^{n\pi} \sqrt{1 + \left(\frac{n^2}{d^2} - 1\right) \sin^2 k} \, dk.$$

As the integrand has period π in k and n is odd, $\int_0^{n\pi} dk = n \int_0^{\pi} dk = 2n \int_0^{\pi/2} dk$, so

$$P = 2Rd \int_0^{\pi/2} \sqrt{1 + \left(\frac{n^2}{d^2} - 1\right) \sin^2 k} \, dk,$$

which can be calculated as an elliptic integral of the second kind,

$$E(a) = \int_0^{\pi/2} \sqrt{1 - a^2 \sin^2 k} \, dk.$$

In Rose 1, $n = 3$ and $d = 5$, and for Rose 2, $n = 3$ and $d = 1$, so the perimeters are

$$P_1 = 10R \times E\left(\frac{4}{5}\right) \quad \text{and} \quad P_2 = 2R \times E\left(\frac{2\sqrt{2}}{3}\right),$$

where, for P_2, we have used the transformation $E\big(\sqrt{-a^2}\,\big) = \sqrt{1 + a^2} E\big(\sqrt{a/(1 + a^2)}\,\big)$. For $R = 19\,\text{cm}$, the values are $P_1 \approx 126.92\,\text{cm}$ and $P_2 \approx 242.5\,\text{cm}$.
