# Supplementary figures and images for: An open-source closed-loop Virtual Reality system to investigate social interactions and collective behavior in fish
Source: PLoS One. 2026 Jan 21;21(1):e0339909. doi: 10.1371/journal.pone.0339909 (PMC12823003; doi:10.1371/journal.pone.0339909)

Occulting  
panels

Depth  
camera

Fish tank

Videoprojector

Mirror

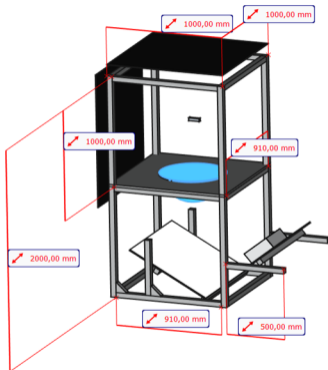

Supplement: S1 Fig — Descriptive diagram (left) and dimensions (right). (PDF) [file pone.0339909.s002.pdf]

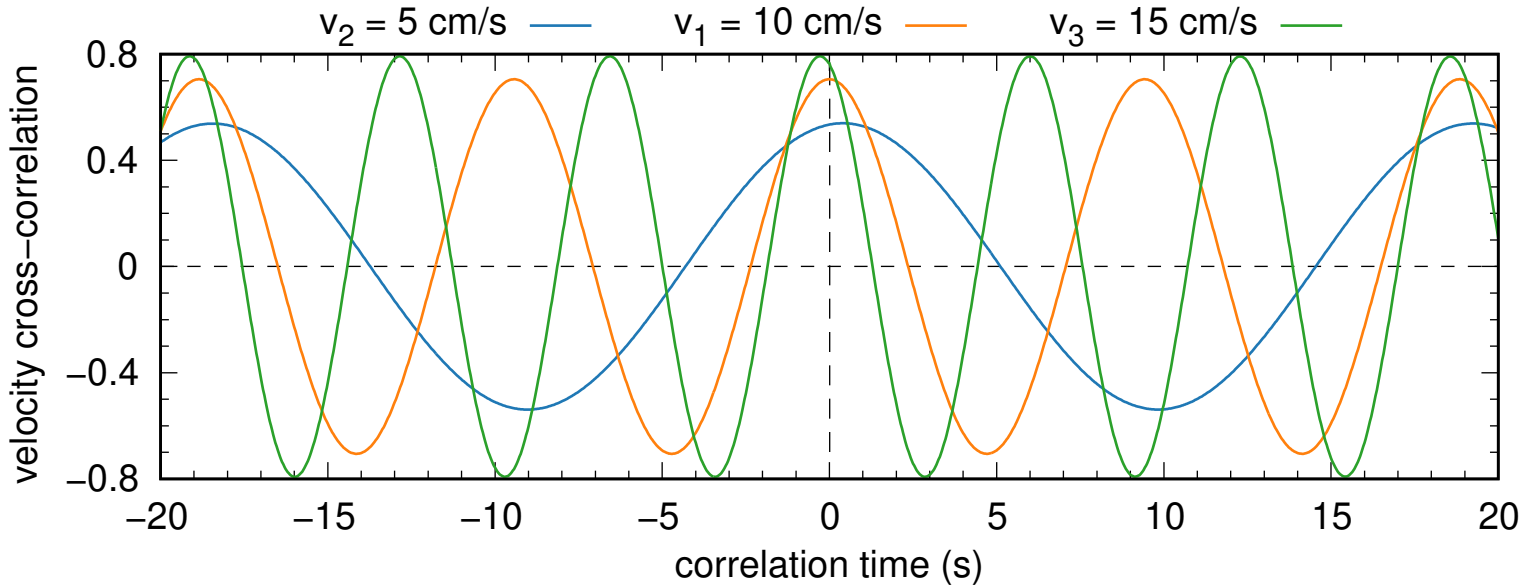

Supplement: S2 Fig — Virtual fish conditions correspond to C1, C2, and C3, with v1=10 (orange), v2=5 (blue), and v3=15 cm/s (green). The trajectories of the virtual fish are circles of radius R = 15 cm at depth z = 5 cm corresponding to a distance to the wall of rw=5.4 cm. Maximum correlation is reached at t1 = 0 s + nT1, t2 = 0.4 s + nT2, and t3 = −0.267 s + nT3, where T1=3π s, T2=6π s, and T3=2π s, for n=0,±1,±2,…. (PDF) [file pone.0339909.s003.pdf]

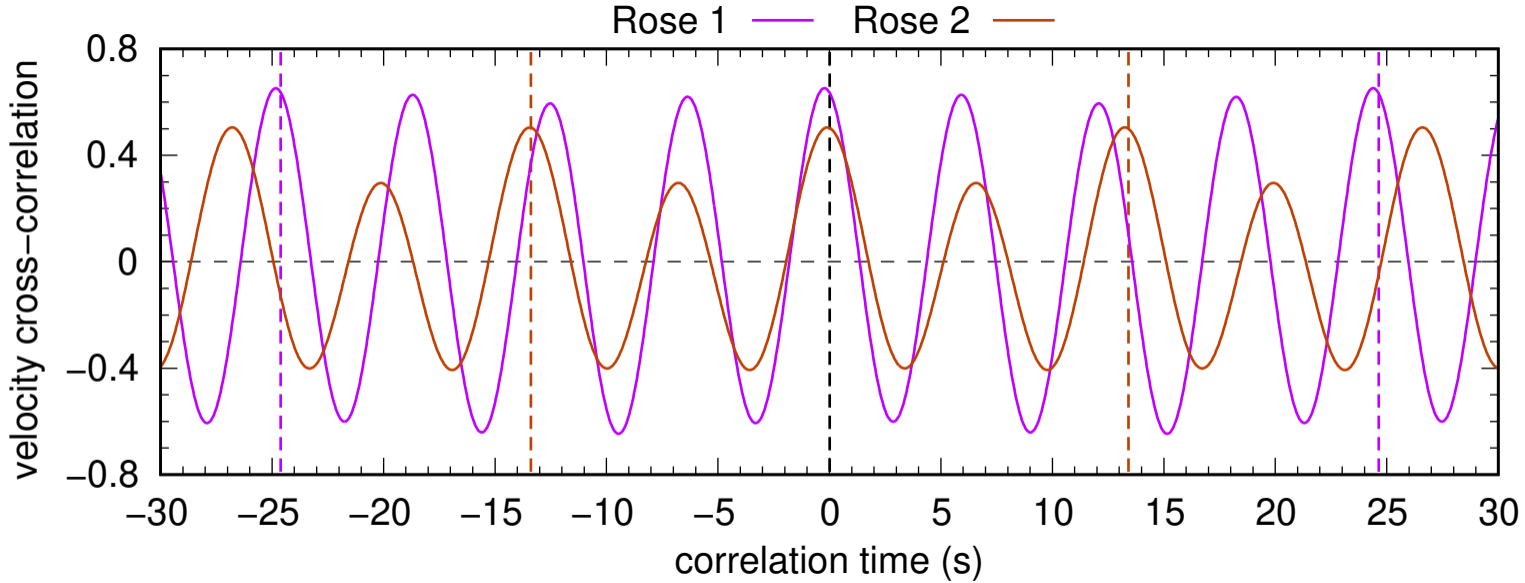

Supplement: S3 Fig — In both cases, the swimming speed is v=10 cm/s, the minimum distance to the wall is rw=1.4 cm, and the swimming depth is z = 5 cm (corresponding to a maximum radius of R = 19 cm). The maximum correlation for Rose 1 (purple) is reached at t1 = −0.23 s + nT1, with T1 = 24.6 s, and for Rose 2 (brown) at t2 = −0.1 s + nT2, with T2 = 13.4 s, for n=0,±1,±2,…. Vertical dashed lines indicate the respective period lengths T1 and T2. (PDF) [file pone.0339909.s004.pdf]
