# Supplementary material for: An open-source closed-loop Virtual Reality system to investigate social interactions and collective behavior in fish
Source: PLoS One. 2026 Jan 21;21(1):e0339909. doi: 10.1371/journal.pone.0339909 (PMC12823003; doi:10.1371/journal.pone.0339909)
Supplement: S1 Table — (PDF) [file pone.0339909.s005.pdf]

**Item Description**	**Quantity**
Aluminum profile 45 × 45 4 slots c - 2 m	4
Aluminum profile 45 × 45 4 slots 10 MM - 1 m / 91 cm	16
Aluminum profile 45 × 45 4 slots 10 MM - 0.5 m	4
Long mounting bracket for 45 × 45 profiles + screw + cover	30
Protective cap for aluminum profiles 45 × 45 10 MM slots	12
Angle bracket for 45 × 45 profiles	8
Center screw for 10 MM slot profile	20
Post-assembly fastening nuts with retaining function for 10 mm slot profiles - M8 Thread	100
Dome-head fastening screw - Thread M8x16 - Hex socket head	100
Washer 18 × 8 × 1.5	100
Blackout acrylic panel 1 × 1 × 1.2 m	3
Blackout acrylic panel 1 × 1 × 1 m	1
Blackout curtain	1
Acrylic bowl - 50 cm diameter - 15 l contenance	1
Depth camera Realsense D435	1
Computer - at least Intel i7 13th gen processor, 32 Gb RAM, Nvidia RTX2080 graphic card	1
4k LED, low latency videoprojector	1
Mirror	1
IR LED lamp with 100W LED heat sink	8
Articulated camera arm	9
