# Supplementary material for: An open-source closed-loop Virtual Reality system to investigate social interactions and collective behavior in fish
Source: PLoS One. 2026 Jan 21;21(1):e0339909. doi: 10.1371/journal.pone.0339909 (PMC12823003; doi:10.1371/journal.pone.0339909)
Supplement: S2 Table — We list the distances obtained for the three conditions compared pairwise. The real fish maintained the same swimming distance and depth relative to the virtual fish across the three swimming speeds tested (i.e., no significant differences were found between the PDFs). However, the swimming speeds of the real fish were significantly different between the three conditions C1, C2, and C3 (i.e., when the virtual fish moved at 5, 10, and 15 cm/s respectively). Values of Hellinger distance are shown in bold font when H > 0.2 (high dissimilarity of the PDFs). (PDF) [file pone.0339909.s006.pdf]

**Observables**	**Conditions** (Mean ± std)			**Hellinger distance**		
	C1	C2	C3	C1|C2	C1|C3	C2|C3
Distance between fish (cm)	9.6 ± 8.9	7.7 ± 7.5	9.7 ± 8.2	0.088	0.076	0.12
Speed of the real fish (cm/s)	9.4 ± 3.6	6.3 ± 3.1	11.7 ± 4.5	**0.333**	**0.262**	**0.484**
Depth of the real fish (cm)	4.4 ± 1.3	4.4 ± 1.1	5.1 ± 1.8	0.074	0.186	0.164
