# Supplementary material for: An open-source closed-loop Virtual Reality system to investigate social interactions and collective behavior in fish
Source: PLoS One. 2026 Jan 21;21(1):e0339909. doi: 10.1371/journal.pone.0339909 (PMC12823003; doi:10.1371/journal.pone.0339909)
Supplement: S3 Table — We list the distances obtained for the three conditions compared pairwise. The swimming distance of the real fish to the virtual fish was significantly smaller when the virtual fish was at 8 cm depth. There was also a significant difference in the swimming depth of the real fish between the three conditions C1, C8, and C9. Values of Hellinger distance are shown in bold font when H > 0.2 (high dissimilarity of the PDFs). (PDF) [file pone.0339909.s007.pdf]

Observables	**Conditions** (Mean ± std)			**Hellinger distance**		
	C1	C8	C9	C1|C8	C1|C9	C8|C9
Distance between fish (cm)	9.6 ± 8.9	7.5 ± 7.6	5.4 ± 5.0	0.118	**0.221**	0.139
Speed of the real fish (cm/s)	9.4 ± 3.6	9.2 ± 3.5	9.4 ± 3.1	0.067	0.113	0.111
Depth of the real fish (cm)	4.4 ± 1.3	3.9 ± 1.1	7.6 ± 1.4	**0.224**	**0.7**	**0.774**
