# Supplementary material for: An open-source closed-loop Virtual Reality system to investigate social interactions and collective behavior in fish
Source: PLoS One. 2026 Jan 21;21(1):e0339909. doi: 10.1371/journal.pone.0339909 (PMC12823003; doi:10.1371/journal.pone.0339909)
Supplement: S4 Table — We list the distances obtained for the three conditions compared pairwise. The swimming speed and depth of the real fish were not influenced by the virtual fish’s proximity to the tank wall. Only when the virtual fish was swimming close to the wall at 2.4 cm in condition C7, the real fish maintained a higher distance in comparison with condition C6, in which the distance to the wall of the virtual fish was much larger (10.4 cm). Values of Hellinger distance are shown in bold font when H > 0.2 (high dissimilarity of the PDFs). (PDF) [file pone.0339909.s008.pdf]

Observables	Conditions (Mean ± std)			Hellinger distance		
	C1	C6	C7	C1|C6	C1|C7	C6|C7
Distance between fish (cm)	9.6 ± 8.9	6.3 ± 5.7	10.1 ± 9.3	0.192	0.078	**0.208**
Speed of the real fish (cm/s)	9.4 ± 3.6	10.1 ± 3.4	8.6 ± 3.6	0.084	0.08	0.154
Depth of the real fish (cm)	4.4 ± 1.3	4.4 ± 1.0	4.4 ± 1.3	0.105	0.101	0.124
