# Supplementary material for: An open-source closed-loop Virtual Reality system to investigate social interactions and collective behavior in fish
Source: PLoS One. 2026 Jan 21;21(1):e0339909. doi: 10.1371/journal.pone.0339909 (PMC12823003; doi:10.1371/journal.pone.0339909)
Supplement: Attachment — Submitted filename: Rebuttal Letter Sanchez_et_al_R1.pdf [file pone.0339909.s012.pdf]

Dear Editor,

Please find attached the revised manuscript *“An open-source closed-loop Virtual Reality system to investigate social interactions and collective behavior in fish”* by Stéphane Sanchez, Ramón Escobedo, Renaud Bastien, Boris Lenseigne, Audrey Denis, Mathieu Moreau, Maud Combe, Andrew D. Straw, Clément Sire, and Guy Theraulaz.

We would like to thank you for the opportunity to revise our manuscript. We also thank the two reviewers for their comments and valuable suggestions for improving our manuscript.

In this revision, we provide a “diff” PDF file highlighting the changes made in the manuscript, which are also detailed in our reply to the reviewers. Here, we summarize the most notable changes motivated by the referees’ comments:

- We integrated new experiments with a non-social control (black sphere), whose results confirm the specificity of social responses.
- We have incorporated additional references (19 to 25) to create a comprehensive and thematically organized Related Work section that more clearly positions our contribution within the existing literature.
- We now explicitly report Hellinger distances in the Results section, when comparing distributions between conditions, providing an inferential basis for our conclusions.
- We have calculated the cross-correlations between the velocity vector of the real fish and the corresponding virtual object, providing an additional quantification of the degree of synchronization and response times.
- We clarified that the present work validates the system in open-loop mode, while full closed-loop tests are ongoing; we added perspectives on scaling the system to multi-fish interactions
- We also revised the title of the manuscript to further highlight the open-source nature of the system. The new title is: *“An open-source closed-loop Virtual Reality system to investigate social interactions and collective behavior in fish.”* We believe this formulation better reflects the fact that all software components required for the VR system are provided as open-source tools, which is a central contribution of our work. This ensures that the system can be widely adopted, reused, and further developed by the research community, thereby maximizing its scientific impact.
- We have made minor improvements to the manuscript (a few typos, refining phrasing for clarity…).

We believe these revisions significantly strengthen the manuscript by expanding the scope of the experimental results and addressing all Reviewer concerns.

Sincerely,

Stéphane Sanchez et al.

Responses to Reviewers

Reviewers have raised a total of 6 points that we reproduce here in *black italicized* text. Our answers are given in blue.

Response to the comments of Referee 1

This article sounds very interesting and impressive. Written in good English language.

#1.1: *However, the only flaw that requires major revision and amendment is the related works regarding this study.*

A: We appreciate the Reviewer's positive assessment and constructive requests. In the revised version of our paper, we have incorporated additional references (19 to 25) to create a comprehensive and thematically organized Related Work section that more clearly positions our contribution within the existing literature.

#1.2: *The Result subheading shown the analysis of the experiment followed by discussion. The methodology section needs to be reorganized prior the result and analysis section.*

A: We have reordered the Methods section before the Results section thus following the PloS One template.

#1.3: *Conclusion section is not found. Is it replaced with discussion?*

A: We added a concise conclusion. We hope these revisions make the manuscript clearer, better contextualized, and easier to navigate.

Response to the comments of Referee 2

This paper presents a major methodological contribution by developing a low-cost, open-source, closed-loop virtual reality system that allows the study of social interactions in fish with unprecedented control. The results convincingly demonstrate that real fish perceive and follow virtual fish as credible conspecifics, adjusting their speed, depth, and relative position in a flexible and robust manner. The strength of the study lies in its experimental rigor, the quality of the quantitative data, and the full transparency of the shared codes and data. However, the statistical analysis would benefit from including inferential tests to formally support comparisons between conditions. Furthermore, while the system allows for visual closed-loop, the behavior of the virtual fish remains pre-programmed in an open loop in this study, thus limiting the exploration of more complex bidirectional interactions. Finally, although the method is powerful for deciphering individual rules, it does not yet address group dynamics involving multiple real individuals.

A: We sincerely thank Reviewer 2 for his/her positive and encouraging assessment of our work. We are pleased that the manuscript was recognized as presenting a major methodological contribution, with rigorous experiments, transparent data sharing, and convincing demonstrations of the ability of real fish to treat virtual fish as credible conspecifics. In the revised manuscript, we have also added a specific measure of the dissimilarity between the PDFs to formally compare behavioral responses across conditions, and the corresponding results are now reported in the Results section. Below we address the two other constructive points raised, and explain how we have revised the manuscript accordingly.

Open-loop behavior of the virtual fish.
We agree that in the initial submission the virtual fish followed pre-programmed trajectories. Our revised manuscript clarifies that this study aimed to validate the platform in open-loop conditions, while emphasizing that the system is designed for full closed-loop experiments. We now highlight ongoing work in which the trajectory of the virtual fish is controlled in real time by three-dimensional interaction models derived from the computational analysis of real fish pairs. These future experiments will allow bidirectional interactions and causal tests of social rules, which we will report in a separate article.

Extension to group dynamics.
We acknowledge that the present study also focused on interactions between one real and one virtual fish. The revised version now explicitly outlines how the system will be scaled to project multiple virtual conspecifics that respond dynamically to each other and to the real fish. This will allow tests of how individuals combine information from

multiple neighbors and how local rules scale to group-level coordination, directly connecting to predictions from recent modeling work (Lei et al., 2020; Wang et al., 2022; Xue et al., 2023}.

***#2.1:** To significantly strengthen the statistical robustness of the evaluation, several additional analyses are possible. The use of non-parametric Kruskal-Wallis tests, followed by Dunn post-hoc tests, would allow for a formal comparison of the distributions of speed, distance, and depth between the different experimental conditions.*

A: To address this point, we have now incorporated an additional subsection entitled *"Statistical Metric for Probability Distribution Comparison"* in the **Data Analyses** section. In this new analysis, we quantify differences between behavioral distributions observed under distinct experimental conditions using the **Hellinger distance**, a widely used and robust statistical metric for comparing probability distribution functions. Specifically, given two normalized distributions, the Hellinger distance provides a bounded measure of similarity ranging from 0 (identical distributions) to 1 (non-overlapping distributions). Small values indicate strong similarity, whereas values greater than 0.2 reveal marked differences. This approach allows us to rigorously assess whether variations in fish responses across experimental manipulations (e.g., changes in speed, depth, or distance to the wall of the virtual fish) correspond to statistically meaningful shifts in the underlying behavioral distributions.

We also clarify in the revised manuscript how these analyses complement the descriptive statistics already presented, thereby reinforcing the robustness of our findings. By adding this inferential framework, we ensure that our conclusions are not only visually convincing but also formally supported by statistical tests.

***#2.2:** In addition, calculating cross-correlations between the speeds and directions of the real and virtual fish would objectively quantify their degree of synchronization and response times.*

A: As suggested, we have calculated the cross-temporal correlations between the velocities of the real and virtual objects (a fish or a black sphere) to provide an additional quantification of synchronization and response times. The results fully confirm our previous conclusions. They can be found, together with the figures, in the following parts of the text:
- In Sec. 3.8.2, we use the formula (11) to calculate the cross-correlation, where the real fish is taken as the reference of C(\tau).
- In Sec. 4.1 and Fig. 6C, showing that the correlation with a virtual conspecific is almost three times larger than with a virtual sphere, showing a clear preference for the social stimulus.

- In Sec. 4.2.1 and S2 Fig., we show that in the three cases with different speeds, the values of the correlation remain high, with the real fish anticipating the virtual one at low speed, behaving closely at intermediate values of the speed, and following the virtual fish with a short delay at high speed.
- In Sec. 4.3 and S3 Fig., for the case of the two rhodonea, correlations are again high. The real fish follows the virtual one more consistently in Rose 1 than along Rose 2, as the correlation is higher and the delay longer and negative in Rose 1, where the correlation is higher and the delay longer (about 0.23 s, negative), whereas in Rose 2 the delay is almost zero but the correlation is weaker.

#2.3: *Finally, the addition of a control condition with a non-social stimulus would ensure that the observed behaviors are indeed specific social responses and not simple reactions to a movement.*

A: We thank the Reviewer for her/his suggestion. We have now included a non-social control stimulus consisting of a black sphere of comparable size to the tested fish. The new results show that fish did not follow or adjust their trajectories relative to the sphere, in sharp contrast to their robust following of virtual conspecifics. This confirms that the observed behaviors are indeed specific to social stimuli and not mere responses to moving objects. The revised manuscript includes these data in the Results section 4.1 (Control condition with a non-social stimulus) and discusses their implications in confirming the social specificity of the responses to 3D avatars.

Cited References :

Xue, T., Li, X., Lin, G., Escobedo, R., Han, Z., Chen, X., Sire, C. & Theraulaz, G. 2023. Tuning social interactions' strength drives collective response to light intensity in schooling fish. Plos Computational Biology, 19(11):e1011636.

Wang, W, Escobedo, R., Sanchez, S., Sire, C., Han, Z. & Theraulaz, G. 2022. The impact of individual perceptual and cognitive factors on collective states in a data-driven fish school model. Plos Computational Biology, 18: e1009437.

Lei, L., Escobedo, R., Sire, C., Theraulaz, G. 2020. Computational and robotic modeling reveal parsimonious combinations of interactions between individuals in schooling fish. Plos Computational Biology, 16: e1007194.
